# Triethanolamine Stabilization of Methotrexate-β-Cyclodextrin Interactions in Ternary Complexes

**DOI:** 10.3390/ijms150917077

**Published:** 2014-09-25

**Authors:** Jahamunna A. A. Barbosa, Ariana Zoppi, Mario A. Quevedo, Polyanne N. de Melo, Arthur S. A. de Medeiros, Letícia Streck, Alice R. de Oliveira, Matheus F. Fernandes-Pedrosa, Marcela R. Longhi, Arnóbio A. da Silva-Júnior

**Affiliations:** 1 Graduate Program on Pharmaceutical Sciences, Department of Pharmacy, Federal University of Rio Grande do Norte (UFRN), Av. General Gustavo Cordeiro de Farias, Petrópolis, 59072-570 Natal, Brazil; E-Mails: jamunauepb@gmail.com (J.A.A.B.); arthurmedeiros@ymail.com (A.S.A.M.); arthurmedeiros@ymail.com (M.F.F.-P.); 2 Research and Pharmaceutical Technology Development Unit (UNITEFA, CONICET-UNC) and Department of Pharmacy, Faculty of Chemical Sciences, National University of Córdoba, Ciudad Universitaria, X5000HUA Córdoba, Argentina; E-Mails: ariana@fcq.unc.edu.ar (A.Z.); alfredoq@fcq.unc.edu.ar (M.A.Q.); alfredoq@fcq.unc.edu.ar (M.R.L.); 3 Graduate Program on Development and Technologic Innovation in Pharmaceuticals, UFRN, Av. Salgado Filho, 3000-Lagoa Nova, 59072-970 Natal, Brazil; E-Mails: polyanne_melo86@yahoo.com.br (P.N.M.); leticiastreck@gmail.com (L.S.); 4 Graduate Program on Healthy Sciences, UFRN, Av. General Gustavo Cordeiro de Farias, Petrópolis, 59072-570 Natal, Brazil; E-Mail: farmalix@yahoo.com.br

**Keywords:** methotrexate, cyclodextrin, inclusion complexes, ternary complexes, drug delivery systems

## Abstract

The interaction of methotrexate (MTX) with beta-cyclodextrin (β-CD) in the presence of triethanolamine (TEA) was investigated with the aim to elucidate the mechanism whereby self-assembly cyclodextrin systems work in association with this third component. Solubility diagram studies showed synergic increment of the MTX solubility to be about thirty-fold. Experiments using 2D ROESY and molecular modeling studies revealed the inclusion of aromatic ring III of the drug into β-CD cavity, in which TEA contributes by intensifying MTX interaction with β-CD and stabilizes MTX:β-CD:TEA ternary complex by electrostatic interaction. The maintenance of these interactions in solid phase was also studied in ternary MTX:β-CD:TEA and comparisons were made with freeze dried binary MTX:β-CD and physical mixtures. FTIR studies evidenced that MTX–β-CD interaction remained in solid ternary complexes, which was also supported by thermal (differential scanning calorimetry (DSC), thermogravimetric analysis (TG)/first derivative of TG analysis (DTG) and C,N,H elementary analysis) and structural (X-ray diffraction analysis, (XRD)) studies, mainly regarding the increment of drug stability. The efficient *in vitro* drug dissolution studies successfully demonstrated the contribution of ternary complexes, which highlights the importance of this possible new raw material for further applications in drug delivery systems.

## 1. Introduction

Methotrexate (2*S*)-2-[[4-[(2,4-diaminopteridin-6-yl) methyl-methylamine] benzoyl] amino] (MTX) is an anticancer drug that inhibits the dihydrofolatereductase enzyme, which interferes in the formation of DNA, RNA and proteins. This drug is also commonly used by oral administration in “psoriasis” treatment, which is a chronic inflammatory disease that causes skin lesion [1,2]. However, due to its short half-life (1.5–3.5 h), a high dosage or frequent administration of the drug is required, causing potential side effects. An alternative to increasing the efficacy of MTX for psoriasis treatment is its application directly to the lesions with consequent drug permeation into the skin, which limits the potential side effects [3].

Limitations regarding topical administration of MTX targeting lesions in psoriasis or skin tumors are well established in literature. A skin and subcutaneous implantable device, based on a protein matrix was purposed for intralesional MTX, in order to improve the local chemotherapy response. However, the drug administration involved an invasive procedure [4]. Thus, some transdermal delivery systems associated or not with iontophoresis for MTX have been proposed to improve its skin permeation. Among them, those that could be highlighted are the microemulsions [5]; ethanolic liposomes [6] and recently the solid-in-oil nanocarriers [7]. However, these nanotechnological drug delivery systems (NDDS) involve oil presence or a refined preparation procedure. Moreover, the low MTX loading in hydrogel vehicles with suitable drug permeation remains unsolved [8]. This occurs because MTX is characterized as a drug that is practically insoluble in water in non-ionized form, which limits its preparation in topical formulations [9].

These NDDS have been developed with the aim to overcome the undesirable properties of drugs. Moreover, these systems may be applied to release the necessary amount of drug to the targeted site for a specific period of time with precision and efficacy. The obtainment of inclusion complexes with cyclodextrins (CDs) is a strategy that has been widely used for NDDS development, due to their ability to alter physical, chemical and biological properties of guest drugs such as solubility, stability, and bioavailability.

The three most common naturally occurring CDs are α-cyclodextrin (α-CD), β-cyclodextrin (β-CD) and γ-cyclodextrin (γ-CD), which are constituted of six, seven and eight units of d-glucopyranose linked by α-1,4 bond into a macrocycle, respectively [10]. Each cyclodextrin can host hydrophobic or water insoluble compounds by a “host–guest” mechanism, which has been used in dermal applications to improve and optimize transdermal delivery of drugs intended for local distribution of drugs in specific skin layers. These inclusion complexes enhance transdermal absorption of drugs because they guarantee a soluble drug fraction in non-ionized form, which improves the diffusion coefficient through skin due to the apparent change in the molecule’s partition coefficient. Thus, these molecules permit the use of suitable aqueous vehicles and avoid undesirable effects associated with systemic use.

β-Cyclodextrin was chosen for the present study due to it being the most common pharmaceutical excipient that is applied to enhance the solubility of drugs. However, this natural cyclodextrin has low solubility in water, which can limit its use in pharmaceutical formulations. Its ability to form inclusion complexes with specific molecules depends on the interaction of the guest molecule with the hydrophobic cyclodextrin cavity in the aqueous environment, which consequently increases the aqueous solubility of the drug. In some cases, a low complexation efficiency of the specific drug would require a larger amount of cyclodextrin, which limits its application in solid or liquid pharmaceutical formulations.

Recently, several strategies using ternary complexes have been used to extend the inclusion capacity of cyclodextrins or to improve the enhancement of aqueous solubility of guest drugs. Basically, these supramolecular aggregates are composed of three different molecular entities involving drug, cyclodextrin and a third component, which is selected with the aim to improve desired physicochemical, chemical or transport properties of a specific drug. The molecular entities selected for this purpose include different substances such as, hydrophilic polymers, amino acids, organic acids (maleic, fumaric, citric acid and l-tartaric acids) and hydroxyl organic amines. Triethanolamine (TEA) certainly has been successfully used with the purpose to improve the efficiency of drug delivery and reduce the CD amount [11,12]. Specifically regarding ternary complexes for topical application, TEA was an effective additive in the improvement of the corneal delivery of acetazolamide and increased the *in vivo* efficacy of drug with a reduction in side effects [12]. However, a diversity of interactions among these three coexisting components is possible and includes potentially synergetic effects, a competitive effect or neutralizing favorable effects of CD. The effects of TEA and the involved mechanism of their interactions in inclusion complex formation remain unclear or misunderstood.

Experiments were conducted considering the possible administration of MTX in a hydrogel NDDS containing ternary inclusion complex system (TC) in the local skin lesions for psoriasis treatment. The work focused initially on assessing the interactions between β-CD and MTX in the presence of TEA and evaluated its ability to enhance drug solubility, which can provide a new solid raw material for MTX. The interactions in aqueous solution were investigated using molecular modeling, phase solubility diagrams and NMR studies. The solid systems were obtained by freeze-drying method and their physicochemical properties were assessed by quantitative analysis, scanning electron microscopy (SEM), Fourier transform infrared spectroscopy FTIR, thermal analysis (differential scanning calorimetry (DSC), thermogravimetric analysis (TG)/first derivative of TG analysis (DTG) and C,N,H elementary analysis), X-ray diffraction analysis (XRD) and *in vitro* drug dissolution studies.

## 2. Results and Discussion

### 2.1. Effect of Triethanolamine (TEA) on Methotrexate (MTX)–Beta-Cyclodextrin (β-CD) Interactions in Aqueous Medium

**Figure 1:**
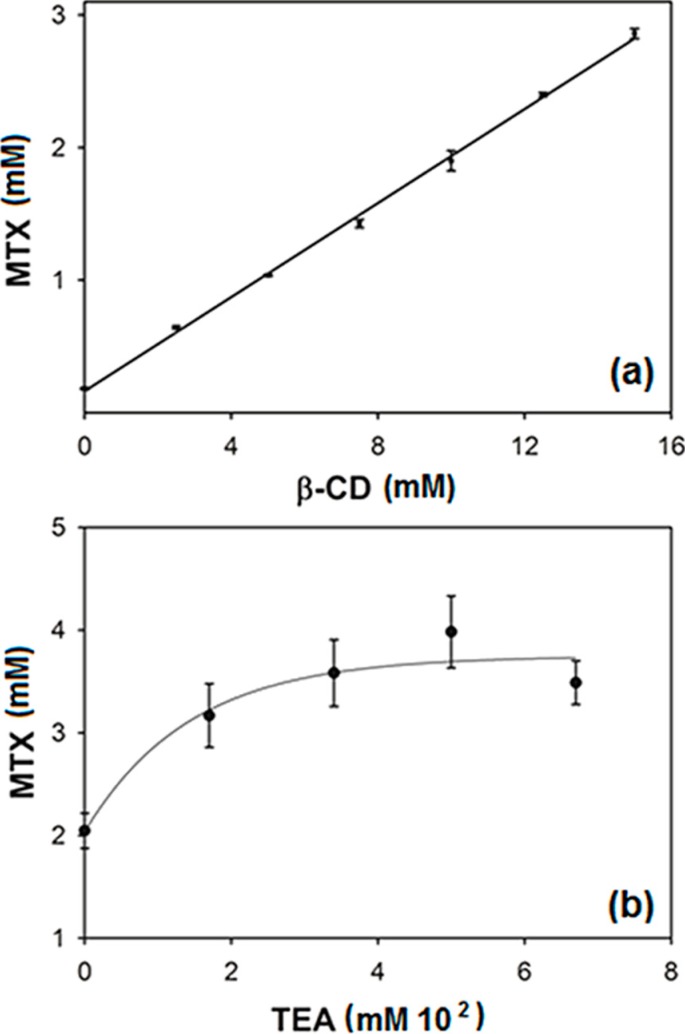
Phase solubility diagrams of (**a**) Methotrexate (MTX) in different β-cyclodextrin (β-CD) concentrations; and (**b**) MTX in fixed 15 mM β-CD associated with different triethanolamine (TEA) concentrations.

The linear relationship between apparent aqueous solubility of MTX and the used β-CD concentration in water is shown in Figure 1a, which was verified by the very high correlation coefficient (*r*= 0.99) observed in the straight line fitted plot (*y* = 0.1772*x* + 0.0002). The linearity was additionally confirmed by one-way ANOVA of experimental data, which demonstrated significant linear regression and no significant linearity deviation. The linear phase diagram suggests the occurrence of soluble complexes with 1:1 stoichiometry and *K*_1:1_ = 1179.8 M^−1^ was calculated by using Equation (1) described in the Experimental Section 3.2. The maximum studied β-CD concentration increased the apparent MTX aqueous solubility about 15 times more than that observed in water. Generally, the total complexation of all cyclodextrin molecules in solution did not occur, but frequently these supramolecular aggregates exhibit a relationship between CD and guest molecule of 1:1 [13,14,15] as seen in the study with hydrochlothiazide and β-CD at different temperatures and pH [16]. In this study, considering the solubilized drug amount and β-CD concentration used, a molar ratio of about 1:5 MTX:β-CD was identified, which was used to prepare solid complexes. The majority of *K*_1:1_ values for binary inclusion complexes ranged from 50 to 2000 M^−1^ [17,18].

In order to increase the effect of β-CD, the solubility of drug was accessed in a β-CD aqueous solution (15 mM) buffered with KH_2_PO_4_, 100 mM (pH = 5.0) containing different TEA concentrations (0 to 0.67 M) (Figure 1b). This TEA concentration range is the range generally investigated for ternary complexes. Drug solubility increments of about 75%, 94% and 70% were observed in the samples containing 0.34, 0.5 and 0.67 M of TEA respectively, which were statistically different (ANOVA followed by *post hoc* Dunnet test, *p* < 0.05) to the value observed for 15 mM β-CD solution. Association of hydrophilic compounds is routinely used to increment the soluble fraction of drug or to reduce the amount of used cyclodextrin [17,18,19]. As previously reported in the literature for other drugs [20,21], a synergic effect was found when using these two solubilizers simultaneously because MTX aqueous solubility was enhanced about 26, 29 and 25 times for cited TEA concentrations. Similar results were obtaining using 0.34 M of TEA with hydropropyl-β-cyclodextrin for sulfisoxazole [21], acetazolamide [12] and flurbiprofen [22,23].

### 2.2. ^1^H-NMR Spectroscopic Studies

**Figure 2:**
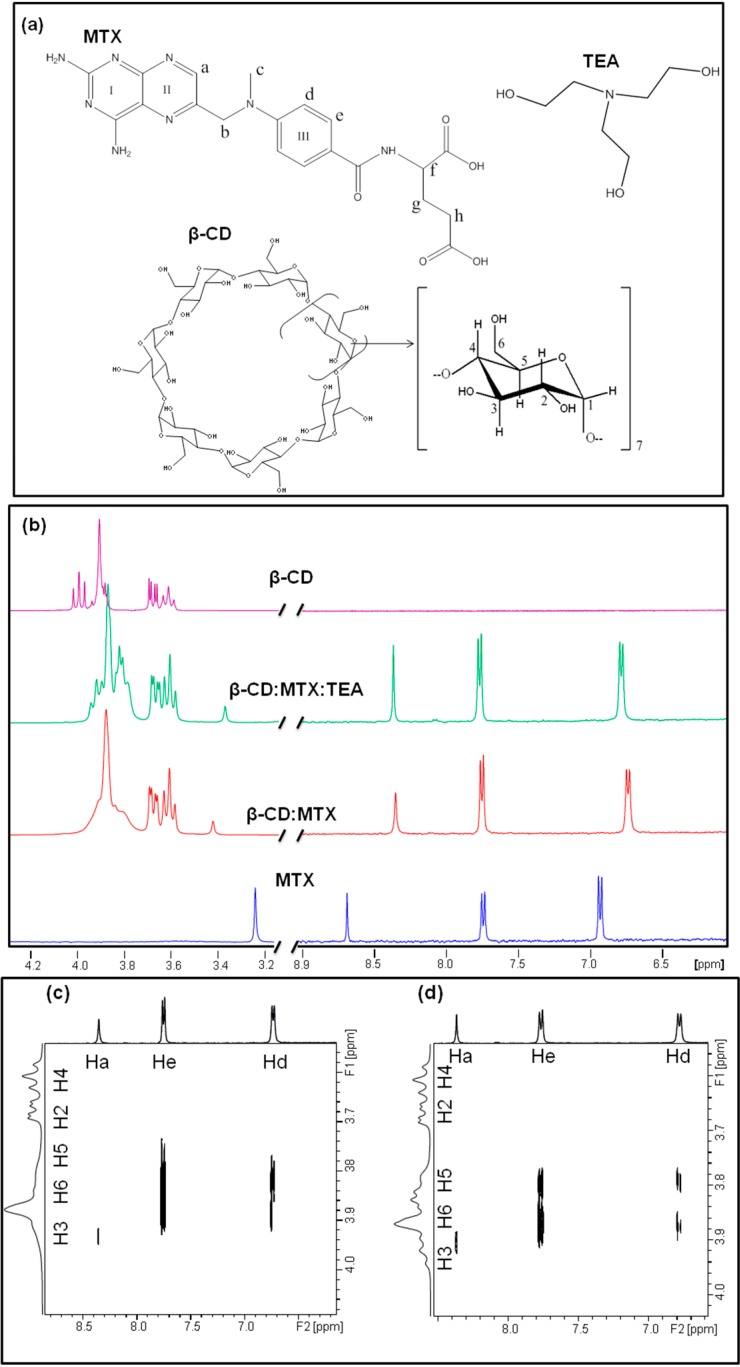
(**a**) Schematic representation of chemical structure and proton atom numbering scheme for MTX, β-CD and TEA; (**b**) ^1^H-NMR Spectra of MTX, binary MTX:β-CD complex, ternary MTX:β-CD:TEA complex and β-CD; (**c**) Partial contour plot of the 2D ROESY spectrum of (**c**) MTX:β-CD binary; and (**d**) MTX:β-CD:TEA ternary complexes (F1 β-CD protons and F2 MTX protons).

^1^H-NMR experiments were carried out in order to study the formation of inclusion complexes MTX:β-CD and MTX:β-CD:TEA. Comparisons among pure components were performed with their corresponding signals in the binary and ternary complexes. As seen in Table 1 and Figure 2b, in the presence of β-CD, almost all MTX proton resonances were modified, and exhibited both upfield and downfield displacements with respect to those of the pure drug. The signals corresponding to β-CD also shed light on the structure of these complexes. A marked upfield displacement for the internal protons of β-CD (H_3_, H_5_ and H_6_, Figure 2a) in both binary and ternary complexes was observed which could be due to the inclusion of groups that are rich in π electrons, such as the aromatic rings of MTX (I, II or III, Figure 2a) in the β-CD cavity. As expected, only small changes in the chemical shifts were observed for the H_1_, H_2_ and H_4_ protons located outside the cavity. ^1^H-NMR experiments confirmed these interactions, but it is difficult to confirm the geometry of the complexes, which can be affected by some factors, such as the stoichiometry of the complex, temperature, and hydrate content of β-CD [24].

**Table 1:** Chemical shifts for protons of MTX and β-CD in different systems.

Studied Protons	Free State (ppm)	Binary Complex (ppm)	Δδ (ppm)	Ternary Complex (ppm)	Δδ (ppm)
MTX					
Ha	8.6892	8.3530	−0.3362	8.3677	−0.3215
Hc	3.2404	3.4216	0.1812	3.3696	0.1292
Hd	6.9292	6.7362	−0.1930	6.7828	−0.1464
He	7.7425	7.7527	0.0102	7.7676	0.0251
β-CD					
H1	5.0984	5.0875	−0.0109	5.0845	−0.0139
H2	3.6778	3.6762	−0.0016	3.6670	−0.0108
H3	3.9936	3.9065	−0.0871	3.9416	−0.0520
H4	3.6118	3.6079	−0.0039	3.6051	−0.0067
H5	3.9063	3.8395	−0.0668	3.8103	−0.0960
H6	3.9063	3.8782	−0.0281	3.8694	−0.0369

The spatial interaction between atoms of the host and guest molecules and corresponding three-dimensional geometry of the complexes was observed through the intermolecular dipolar cross-correlations accessed in 2D ROESY experiments. The ROE is a two-dimensional technique based on the Nuclear Overhauser Effect (NOEs), in which cross-peaks may be observed between protons if the corresponding internuclear distance is smaller than 3–4 Å. An expansion of the ROESY spectrum obtained for binary (Figure 2c) and ternary complexes (Figure 2d) shows the interaction between aromatic protons of MTX (H_d_ and H_e_) and H_3_, H_5_ and H_6_ protons of β-CD, indicating that the aromatic ring III of the drug is deeply internalized in the cyclodextrin cavity in both binary and multicomponent complexes. In addition, H_a_ protons of MTX only exhibited correlations with the H_3_ proton, indicating that it is located closer to the wide rim of the molecule torus.

### 2.3. Molecular Modeling Studies

**Figure 3:**
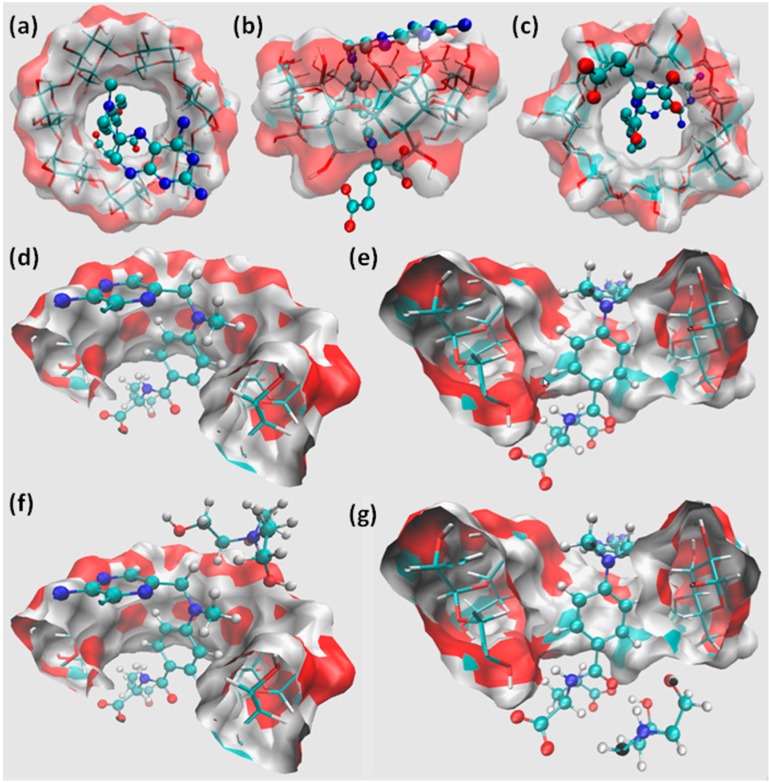
Binding mode predicted by molecular docking assays of MTX to β-CD in (**a**) top view; (**b**) side view; and (**c**) rear view; (**d**) MTX:β-CD (*cluster 1*); (**e**) MTX:β-CD (*cluster 2*); and clusters of MTX:β-CD:TEA; (**f**) *pose-1* and (**g**) *pose-2*.

The docking of the ligand (MTX) to β-CD was performed by considering its ionization state at pH = 5.0, with a single binding mode found in different views (Figure 3a–c). The scoring value obtained as assigned by the *ChemGauss3* force field was −35.78, with the central aromatic ring (region A) inserted into the β-CD hydrophobic cavity, the 2,4-diaminopteridin ring (region B) oriented towards the wider cavity of the host molecule and the pentanedioate (region C) of MTX oriented towards the narrower rim of β-CD. This predicted binding conformation is consistent with the upfield displacements observed by ^1^H NMR, in which a high shielding effect on H_3_ and H_5_ is produced by the benzyl ring. Usually, the inclusion complexes involve the interaction of the hydrophobic cavity of β-CD with aromatic moiety of the guest molecule (when it is present), which is hydrophobic in nature, whereas the other groups remain outside of the hydrophobic zone of β-CD molecule, forming hydrogen bonds with the surrounding water molecules as well as with the hydroxyl groups of the CD molecule [25,26,27,28].

The inclusion complexes shown in Figure 3 were afterwards subjected to assays of molecular dynamics, observing that the inclusion complex conformation was maintained throughout the 10 ns of simulation when explicit solvent conditions and temperature were applied to the system. Table 2 shows the energetic decomposition analysis performed over the whole simulated trajectory, in which it can be seen that a high affinity of MTX for β-CD is predicted, with an estimated *∆G* of −19.50 kcal·mol^−1^. This affinity results from a significant stabilization derived from both van der Waals contacts (−33.20 kcal·mol^−1^) and electrostatic (hydrogen bond, ion–dipole, dipole–dipole) interactions (−30.07 kcal·mol^−1^) between MTX and β-CD. These interactions are already established in the literature as the main driving forces responsible for the formation of the CD inclusion complexes [26,29].

**Table 2:** Energetic decomposition analysis (MM-PBSA method) performed on the molecular dynamics trajectory for MTX:β-CD and MTX:β-CD:TEA complexes.

Energetic Component	Value (kcal·mol^−1^)	
	Binary Complex	Ternary Complex
Electrostatic	−30.07	−220.22
Van der Waals	−33.20	−30.09
Total Gas Energy	−63.27	−250.30
Solvation Energy	43.76	217.87
Estimated ∆*G*	−19.50	−32.43

The persistence of intermolecular interactions between MTX and β-CD were further studied over the whole trajectory, and, as can be seen in Table 3, the stabilization derived from the electrostatic component is mainly originated in hydrogen bonds formed between the O-H_6_ from β-CD with the di-ionized carboxylic acid moieties from MTX.

**Table 3:** Intermolecular MTX-β-CD interactions with occupancy (%) in the overall trajectory and average distance (Å) are informed.

Atoms	Occupancy (%)	Average Distance (Å)
MTX(O_3_):β-CD (O-H_6_)	24.54	2.68 (±0.11)
MTX(O_4_):β-CD (O-H_6_)	15.80	2.65 (±0.15)
MTX(O_2_):β-CD (O-H_6_)	5.52	2.70 (±0.12)
β-CD (O_3_):MTX (NH2_8_)	6.35	2.88 (±0.08)

In order to understand the MTX:β-CD:TEA interactions, the MD trajectory obtained from the complex between MTX and β-CD was subjected to a clustering analysis based on the root mean squared deviation (RMSD) of the corresponding structures. Two main clusters were obtained (Figure 3d,e), and they were afterwards used to perform the molecular docking of TEA. In this way, the ternary complex was predicted by molecular docking, with two binding poses being obtained for TEA: the first, named *pose-1* (Figure 3f) corresponded to the conformation in which the third component is positioned towards the wide rim of β-CD, establishing hydrogen bond contacts with the 2-OH and 3-OH of the macromolecule. The second binding pose was identified as *pose-2*, which exhibited a significantly different binding mode of TEA (Figure 3g), in which this third component is positioned towards the narrower rim of β-CD. In this conformation, ionic interactions between the positively charged amine group of TEA and the negatively charged carboxyl moiety of MTX are clearly driving the positioning of the third component. Additional hydrogen bonds are also established between the hydroxyl groups of TEA and the carboxyl moiety of MTX.

Simulations of molecular dynamics show that *pose-1* was not stable under the simulated conditions, observing that TEA dissociated from β-CD, and thus it can be inferred that this ternary complex is not formed. On the other hand, the *pose-2* predicted by molecular docking was stable throughout the simulated trajectory, when considering the above mentioned electrostatic interactions were maintained, and was subjected to energetic decomposition analysis performed over the whole simulated trajectory (Table 3).

We observed previously that TEA competes with benznidazole for β-CD cavity, in which TEA contributed to reducing the drug–CD interaction compared with referred binary complex [30]. In contrast, the present study showed that TEA increased significantly MTX affinity for β-CD when regarding an electrostatic interaction (−220.22 kcal·mol^−1^) that was not observed for the binary complex. On the other hand, the stabilization that originated in the van der Waals interactions was not different when comparing the binary or ternary complex, which suggests that no significant change in the inclusion mode occurs as a consequence of adding TEA. As expected, high desolvation energy is required to establish the corresponding electrostatic interactions (217.87 kcal·mol^−1^), which in turn originates a higher total interaction energy than that obtained for the binary inclusion complex (−32.43 and −19.50 kcal·mol^−1^, respectively). The higher affinity founded for the MTX:β-CD:TEA complex (*pose-2*) helped explain experimental observations derived from the solubility phase studies, in which an almost thirty-fold increase in the solubility of MTX was observed in the presence of 0.17–0.67 M of TEA, confirming the formation of ternary complexes with β-CD.

The confirmation of obtainment of binary and ternary complexes in aqueous medium using phase solubility, molecular dynamics and NMR studies supported the amount of information needed to obtain these complexes in solid phase through freeze-drying and to investigate the interactions among compounds in solid phase.

### 2.4. Drug-Loading Analysis and FTIR Studies

MTX was efficiently charged in the binary and ternary complexes with drug loading of 7.3% ± 0.01% and 8.4% ± 0.3% respectively. The analytical percentage of nitrogen identified in the elementary analysis for different samples (Table 4) confirmed that MTX was fully loaded in solid complexes. 

**Table 4:** C,H,N elementary analysis results.

Sample	Theoretical %N from MTX	Analytical Content (%)		
		C	H	N
MTX	24.65	46.67	5.45	21.65
β-CD	–	38.01	6.78	0.01
β-CD:MTX	1.75%	38.96	6.71	1.55
β-CD:MTX:TEA	1.80%	40.65	6.77	2.74

The higher %N and smaller %C identified for ternary complexes occurred due to the TEA (Figure 2). An analytical %H of about 5.45% was identified for pure MTX, which is different to the theoretical value for anhydrous drug (4.9%), which suggests the possible presence of a hydrate form of drug. This information was clarified thoroughly through TG and XRD analysis.

**Figure 4:**
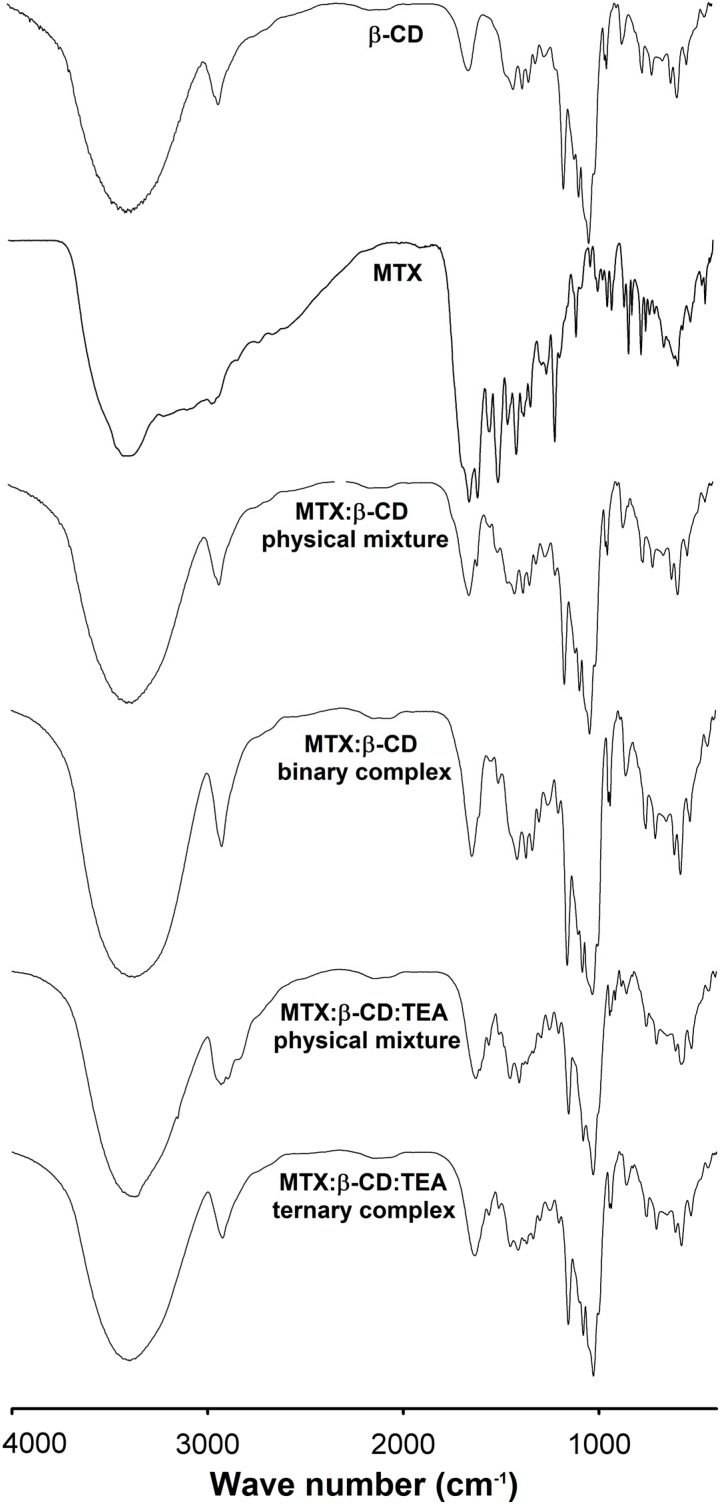
FTIR spectra of pure β-CD and MTX, physical mixtures and respective binary and ternary freeze-dried complexes.

Any interaction involving chemical binding or intermolecular bind interactions among the components was investigated by using FTIR which was carried out for pure components, physical mixtures and drug loaded freeze-dried complexes (Figure 4). The FTIR spectra of β-CD showed characteristic bands of primary and secondary OH groups, intra or inter-molecularly bonded, in the range of 3500–3200 cm^−1^. In this spectral region water molecules (interstitial and intracavity) can also be revealed. The C–H stretching of sp^3^ carbons was identified in the range of 3000 cm^−1^, C–O stretch in the region of 1200–1000 cm^−1^, the cyclic ether group can be observed by the C–O–C stretching in the region of 1150–1085 cm^−1^. The FTIR spectra of MTX showed the characteristic bands of functional groups of carboxylic acid C=O stretching of about 1706 cm^−1^, C–O stretch in the range of 1320–1210 cm^−1^ and O–H stretch in the range of 1440–1395 cm^−1^. The primary amine N–H_2_ stretches were observed between 3400 and 330 cm^−1^, and C–N stretch was observed in region of 1342–1266 cm^−1^. The tertiary amide CO–N–H stretch was observed in the region of 1650–1515 cm^−1^ and aromatic C=C stretch in 1450, 1500 and 1550 cm^−1^.

The FTIR spectra of the physical mixtures β-CD:MTX and β-CD:MTX:TEA presented just an overlapping of pure components, without any relevant difference when compared with each of the pure components. Whereas, FTIR spectra of binary and ternary complexes showed some differences, mainly because of the intensity of tertiary amide CO–N–H (1650–1515 cm^−1^), with bands in the region of between 600 to 950 and 1000 to 1300 cm^−1^.Some studies have described a predominance of stretch of β-CD functional groups in IR spectra of solid complexes, giving evidence of inclusion complexation of the drug [31,32]. The inclusion of MTX in β-CD cavity, as previously demonstrated in NMR and molecular modeling studies, changed the involved energy absorption in the referred IR region. The presence of new hydrogen bonding was verified through OH band broadening, when compared to the spectra of complexes with respective physical mixtures. In addition, comparisons between IR spectra of binary and ternary complexes made it possible to observe different intensities in these same absorption regions and also mainly different amide CO–N–H stretch (1650–1515 cm^−1^) and different aromatic C=C stretch (1450 to 1600 cm^−1^), which also point to the inclusion of this part of the molecule in β-CD cavity and suggest the influence of TEA in these intermolecular interactions. These results are in agreement with 2D ROESY and molecular modeling experiments, which demonstrated the maintenance of the aromatic ring III of MTX deeply included in the β-CD cavity.

### 2.5. Thermal and Structural Studies

**Figure 5:**
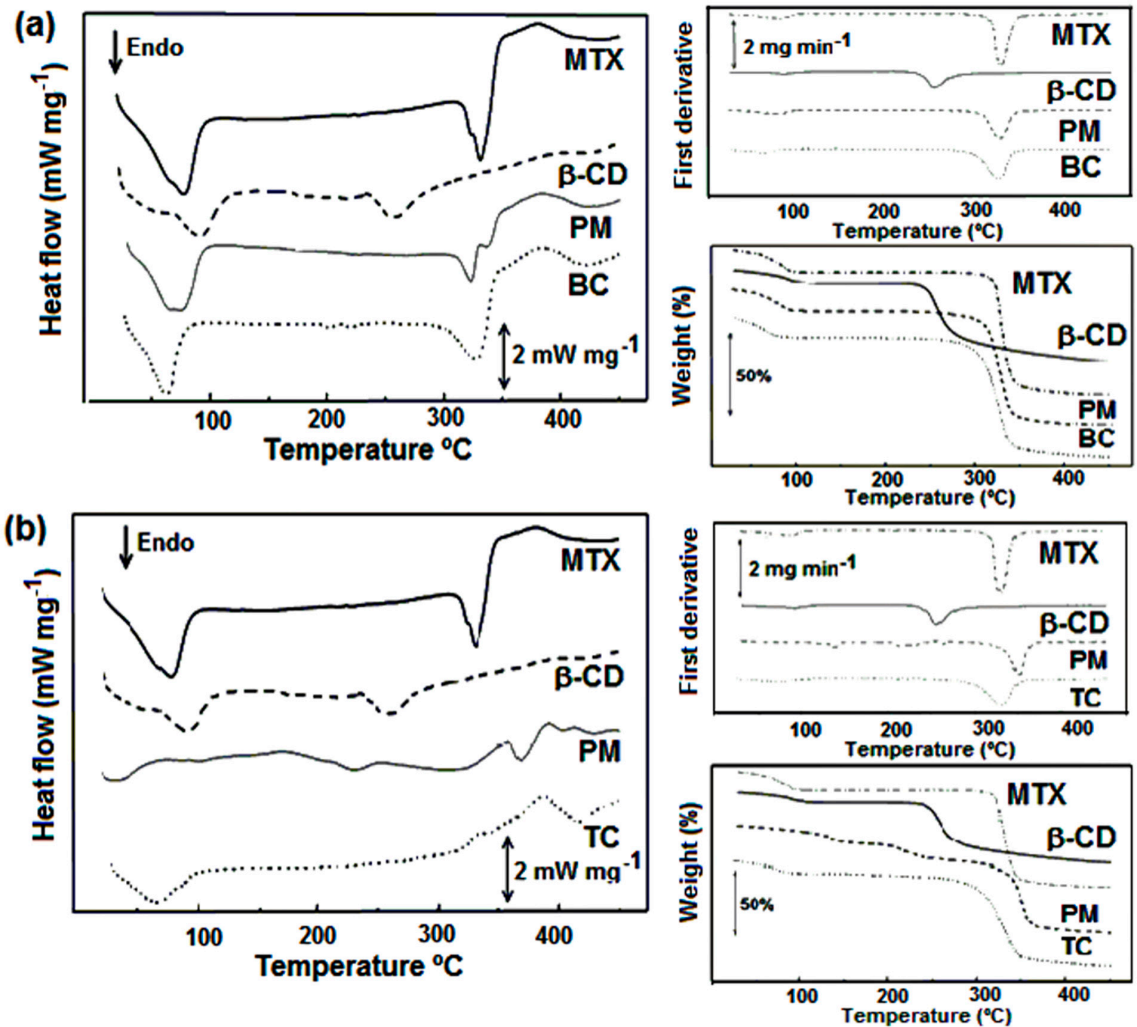
DSC and TG/DTG curves of β-CD, MTX, binary and ternary physical mixtures and binary (BC) and ternary (TC) freeze-dried complexes. Comparisons made for (**a**) binary complexes; and (**b**) ternary complexes.

Figure 5 shows DSC and TG/DTG curves for different samples. In the TG/DTG curves of β-CD, the water evaporation was observed between 63 and 114 °C (Δ*m*_1_ = 13.69%), which corresponded to the endothermic event observed in DSC curve at the same temperature range. The second thermal decomposition event (Δ*m*_2_ = 74.26%) started at about 290 °C. In DSC curve, a sequence of three exothermic-endothermic-endothermic peaks was also observed respectively in the range of 217–230 °C, without weight loss in TG/DTG curves. These events have been mentioned by other authors in the range of 210–240 °C [33]. The mechanism involved with these phase transitions remains unclear, but seems to be associated with crystal hydration during the synthesis of β-CD, which can present different crystalline forms with possible transition among them [33]. The prevalent pseudopolymorphic forms for β-CD crystals are decahydrate or dodecahydrate forms. The water weight loss (13.69%) made it possible to calculate ten water molecules, suggesting the presence of decahydrate in the samples. The structural differences of these hydrates depend on the amount of water molecules and how they are arranged within the CD. This explains melting and thermal decomposition of the β-CD in temperatures above 300 °C, and is mainly regarding the absence of a clear melting peak before the decomposition.

In TG/DTG curves of MTX, the water loss occurred in the range of 84–120 °C (Δ*m*_2_ = 7.6%), suggesting the presence of a hydrate equivalent to two water molecules. This endothermic event (Δ*H* = 71 J·g^−1^) was observed in the DSC curve in the same temperature range. However, some author shave described the identification of two possible hydrate forms such as trihydrate and tetrahydrate [34,35,36]. An endothermic phase transition was observed in the range of 163–239 °C, which was attributed to the melting of a crystalline form, which was followed afterwards by thermal decomposition. Previous studies with MTX-loaded spray dried microparticles reported thermal stability until 200 °C [37].

Comparisons were made for both binary complexes (Figure 5a) and ternary complexes (Figure 5b). The characteristic endothermic events of drug and β-CD were observed in the physical mixture and binary complex. The first event occurred due to the dehydration of samples and the second due to thermal decomposition. However, the cyclodextrin increased the degradation temperature, which occurred above 308 °C for physical mixture and 299 °C for binary complex. This can be explained by the interaction between MTX and β-CD, suggesting the maintenance of inclusion complexes formed in solution after freeze-drying. In addition, the thermal stability associated with absence of significant signal of free β-CD or drug suggests the inclusion complex formation of the majority of drug molecules with possible additional amorphization of material during the preparation procedure. The slight changes observed in dehydration can be attributed to water molecule displacement from β-CD cavity during drug complexation [38].

Thermal behavior of both ternary physical mixture and freeze-dried ternary complex (Figure 5b) also demonstrated an increment in the drug thermal stability in both samples. The different level of TEA molecule dispersion into samples could be observed when comparing ternary complexes with physical mixtures. A weight loss event occurred with DTG peak at about 263 °C with Δ*m* = 9.80%, and can be attributed to TEA evaporation. These experimental data strongly suggest that binary and ternary complexes were successfully prepared by freeze-drying during which cyclodextrin increases the thermal stability of MTX in different studied complexes.

**Figure 6:**
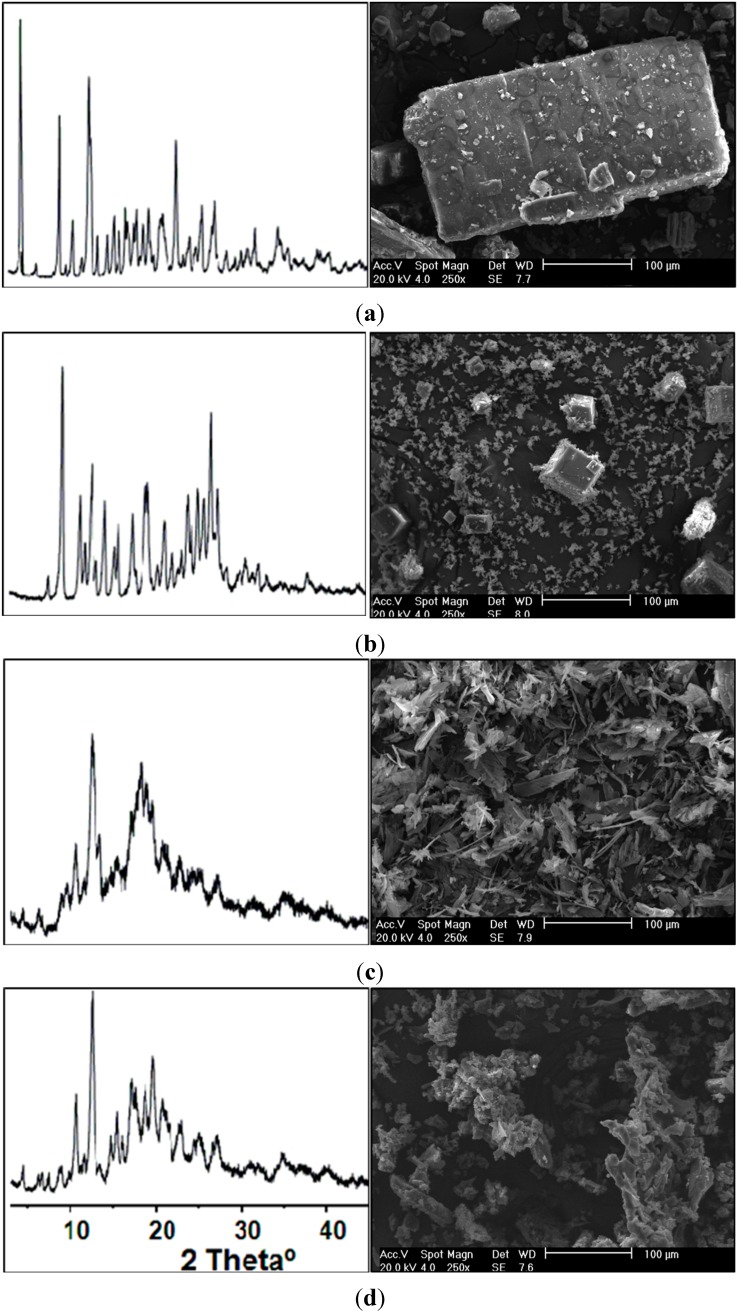
Scanning electronic microscopy (SEM) and X-ray diffraction (XRD) patterns of (**a**) β-CD; (**b**) MTX; (**c**) MTX:β-CD binary; and (**d**) MTX:β-CD:TEA ternary freeze-dried complexes.

The shape and structure of particles were assessed by SEM and XRD respectively (Figure 6). Visually uniform yellow dry powders with the small particle size of methotrexate-loaded β-CD freeze-dried complexes were produced successfully. In the β-CD images (Figure 6a) characteristic parallelepiped forms of crystals were observed [39], which was confirmed by characteristic peaks of crystalline β-CD at 4.4°, 8.9°, 12.3° and 22.6° identified in the XRD pattern (Figure 6a). The smaller signs at higher 2θ values can be considered typical of a cage-type lattice arrangement, which is routinely observed in experiments due to the random packing arrangement of β-CD [38,40].

SEM image of MTX powder (Figure 6b) suggests the simultaneous presence of aggregates with very small particles in amorphous and crystalline states. Similar symmetric crystals with tetragonal structures were also observed, indicating different organization states of drug in the sample. Previous studies reported the identification and characterization of different crystalline habits of MTX involving polymorphism and pseudopolymorphism, mainly regarding powders obtained by crystallization using different solvents [41]. The presence of some MTX tetragonal crystals was confirmed by XRD analysis (Figure 6b). An anterior study performed by Hak-Kim and Gonda [34] described two different crystalline habits of a stable pseudopolimorphous (hydrate) with tetragonal crystalline form. The water content of these two hydrates indicated MTX crystals likely to be dihydrate (about 7.3 wt % H_2_O) and/or trihydrate (about 10.6 wt % H_2_O). Previous studies reported the presence of the tetrahydrate form [36]. The number of water molecules into unit cells could possibly range from two to four molecules in available commercial MTX [35]. In addition, thermal analysis indicated a characteristic dehydrate form with water loss at about 7.6 wt %, which was confirmed by XRD due to the characteristically well-defined diffraction peaks at 7.66 and 9.24, respectively [34].

In SEM images of binary and ternary complexes (Figure 6c,d) crystals of drug or cyclodextrinwere not observed, which confirms obtainment of amorphous complexes [42]. This information can be confirmed in the respective XRD patterns of both binary β-CD:MTX (Figure 6c) and ternary β-CD:TEA:MTX freeze-dried complexes (Figure 6d), in which no characteristic diffraction peaks of crystalline MTX or β-CD were observed. Moreover, amorphization halos were identified for two solid complexes, which suggest that MTX is included into β-CD cavity or dispersed as an amorphous state in these solid dispersions [31,39,43,44].

### 2.6. In Vitro Drug Release Studies

**Figure 7:**
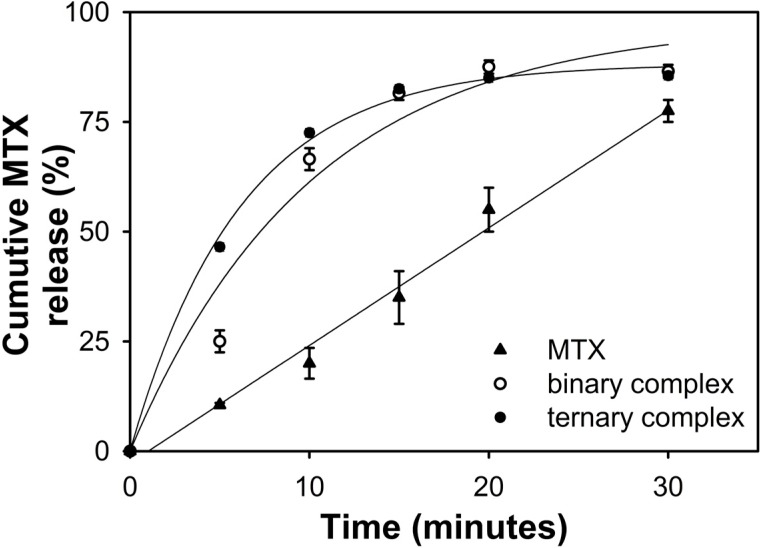
Dissolution studies for MTX, MTX:β-CD binary and MTX:β-CD:TEA ternary complexes.

The type of crystalline state of drug inside solid materials is very important, mainly regarding the polymorphism. The drug distribution in particles is a parameter that may always be considered in the NDDS development, because it directly correlates with drug release rate and consequently drug bioavailability. The maintenance of inclusion complexes in solid phase certainly leads to an interesting *in vitro* dissolution profile. Thus, the dissolution studies were performed for pure MTX and for both binary and ternary freeze-dried complexes with the aim to evaluate the effect of complexation in the drug dissolution rate (Figure 7).

The phase solubility studies demonstrated previously that the maximum β-CD concentration leads to an increase in MTX aqueous solubility of about 15 times and that the presence of TEA raised this effect. When regarding *in vitro* dissolution of drug from binary and ternary complexes in solid phase, a slight difference in slope of drug dissolution profile for ternary complex demonstrated its best performance (Figure 7). This effect was clarified when the amount of MTX dissolved at five minutes was observed (about 45%), which was almost two times faster than binary complexes. A clearly faster dissolution of drug in binary and ternary freeze-dried complexes was observed. Thus, the dissolution performance of ternary complex was superior and the experiments of MTX dissolution performance in different samples demonstrated the success of ternary freeze-dried complex.

## 3. Experimental Section

### 3.1. Materials

MTX, (2*S*)-2-[[4-[(2,4-diaminopteridin-6-yl) methyl-methylamine] benzoyl] amino] (MTX) was purchased from DEG^®^ (São Paulo, Brazil); β-CD was a gift from Roquette^®^ (Labonathus, Brazil); triethanolamine was purchased from Synth^®^ (Labsynth, São Paulo, Brazil). All other reagents were analytical grade. The purified water (1.3 µS) was prepared from reverse osmosis purification equipment; model OS50 LX, Gehaka (São Paulo, Brazil).The D_2_O 99.9 atom%D used in spectroscopic studies was purchased from Sigma–Aldrich (St. Louis, MO, USA).

### 3.2. Phase Solubility Studies

An excess of MTX was hermetically enclosed in flasks containing β-CD aqueous solutions in different concentrations (0 to 15 mM) at 25 ± 2 °C during 72 h, they were subsequently shaken in an ultrasonic bath for 15 min, every 12 h [20]. Sequentially, solutions were filtered through 0.45 µm membranes of cellulose acetate, the pH was measured, remaining at about 4.5 and 5.0 and MTX concentration was analytically determined using the UV–Vis spectrophotometric method previously validated (data not shown). The mean apparent stability constant (*K*_1:1_) was estimated using the slope of linear regression from phase solubility diagrams (*n* = 3), assuming 1:1 M stoichiometry, according to Equation (1), in which So is the solubility of the pure drug in water and the “slope” is the slope of the solubility diagram.

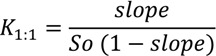
(1)

The effect of TEA (0 to 0.67 M) on drug complexation was investigated in the presence of β-CD (15 mM). The solutions were buffered using 0.1 M KH_2_PO_4_ (pH = 5.0), due to the alkalinizing effect of TEA.

3.3. ^1^H-NMR Spectroscopic Studies

All experiments were performed on a Bruker Avance II High Resolution Spectrometer (Bruker Corporation, Ettlingen, Germany), equipped with a Broad Band Inverse probe (BBI) and a Variable Temperature Unit (VTU). All experiments were conducted at 298 K, using 5 mm sample tubes. The NMR data was processed with the Bruker TOPSPIN 2.0 software (Bruker Corporation, Rheinstetten, Germany). The D_2_O 99.9 atom%D used in spectroscopic studies was purchased from Sigma–Aldrich, ^1^H-NMR spectra were obtained at 400.16 MHz. The chemical shifts (δ) were reported as ppm, and the residual solvent signal (4.80 ppm) was used as the internal reference. Induced changes in the ^1^H-NMR chemical shifts (Δδ) for MTX and β-CD originating from their complexation were calculated according to the following equation: Δδ = δ_complex_ − δ_free_. NMR spectra of pure MTX and their combinations with β-CD and β-CD:TEA were taken in D_2_O.

The geometry of the inclusion complex was studied by two-dimensional Rotating frame Overhauser experiments (2D ROESY). The pulse sequence was: roesygpph19; 2D ROESY with continuous wave spinlock for mixing; phase sensitive and water suppression using a 3-9-19 pulse sequence with gradients; p15 (f1 channel), pulse for ROESY spinlock (200,000 µs); d1:2 s, d19 (delay for binomial water suppression), d19 = (1/(2 × d)), d = distance of next null (in Hz) use gradient ratio:gp 1:gp 2 (30:30); for *z*-only gradients: gpz1: 30%; gpz2: 30% use gradient files; gpnam1: SINE.100, gpnam2: SINE.100. Before Fourier transformation, the matrix was zero, filled to 4096 (F2) by 2048 (F1), and Gaussian apodization functions were applied in both dimensions.

### 3.4. Molecular Modeling Studies

The initial structure of MTX was built using the Gabedit software [45], after conformational and energetic analyses performed with the Gaussian03 software (Gaussian Inc., Wallingford, WA, USA). The corresponding minimum energy conformation was obtained by applying a systematic conformational search based on semi-empirical (AM1) methods, with the final minimum energy conformation optimized by using an *ab initio* (HF-6-31G*) method. The restrained electrostatic potential (RESP) fitted charges model was applied to the final optimized geometry, which was also used to model the electrostatic interactions between MTX and β-CD.

Complexes between MTX and β-CD under different simulated conditions were predicted by molecular docking, using software packages developed by Open Eye Scientific Software [46]. Initial structures of the ligands were obtained as previously mentioned, while the structure of β-CD was retrieved from the Cambridge Structural Database (code: BCDEXD10). The molecular docking procedures consisted of three phases: (a) ligand conformer library generation and parameterization, which was performed using OMEGA software [47] by assuming an energy threshold of 10 kcal/mol; (b) the docking assay, which was performed by applying a fast rigid exhaustive docking approach as implemented in the FRED3 and the OE Docking suite [47,48]. The ChemGauss3 force field was used to score HCT binding to β-CD, with the best ten docked poses being reported; (c) visualization and analysis were carried out using the VIDA v.4.2.1 software [49], selecting the corresponding docking poses that were then subjected to further molecular dynamic analyses.

The Amber12 software package was used for the molecular dynamic (MD) studies [50]. Atomic charges and molecular parameters corresponding to HCT were assigned from the GAFF Force Field [51], while those corresponding to β-CD accounted for the GLYCAM_06 force field [52]. To perform these simulations, the complexes obtained by molecular docking were used as initial structures, solvated with a pre-equilibrated TIP3P cubic box of explicit water molecules, and subjected to minimization. The minimized systems were then heated to the target temperatures (298, 310 and 318 K) for 20 ps, using a time step of 2 fs under constant pressure and temperature conditions. The SHAKE algorithm was applied to constrain bonds involving hydrogen atoms. After heating the system, an equilibration phase (1 ns) was performed followed by the corresponding production phase (10 ns).

Analyses of the MD trajectories were carried out using the Cpptraj module of Amber12, with energetic decomposition analyses being performed by applying the Molecular Mechanics Poisson–Bolzmann Surface Area (MM-PBSA) approach [53]. The resulting trajectories were visualized through VMD v.1.9 software [54]. Molecular dynamic trajectories were obtained from Compute Unified Device Architecture (CUDA) designed code (pmemd.cuda), with computational facilities provided by the GPGPU Computing group [55] at the Faculty of Math, Astronomy, and Physics (FAMAF), National University of Córdoba, Argentina.

### 3.5. Solid Samples Preparation

Solid binary MTX:β-CD complex (BC) was prepared by freeze-drying method. The drug/cyclodextrin ratio was established from the experimental data from solubility diagrams, using the amount of drug solubilized in the presence of β-CD at 15 mM. Thus, suitable amounts of each component were dispersed in water, and shaken in an ultrasonic bath at 25.0 ± 0.1 °C constant water temperature until the drug was dissolved completely to obtain MTX:β-CD with 1:5 molar ratio. Solutions were frozen at −80 °C for 24 h to ensure complete freezing before the freeze-drying (Freeze Dye Liobras model L202, São Carlos-SP, Brazil). For ternary complexes (TC) preparation, 10% *w*/*w* of TEA in relation to amount of drug and cyclodextrin was added to the solutions. Physical mixtures were prepared using the same ratio with the components by uniformly mixing it in a mortar.

### 3.6. Physicochemical Aspects of Freeze-Dried Complexes

Shape and aspect of surface of freeze-dried complexes and pure components were investigated through scanning electronic microscopy (SEM) (ESEM, XL 30 Philips, Cleveland, OH, USA). The particles were dried and mounted on metal subs using double-sided adhesive carbon tape with conductive effect and then coated with a thin layer of gold in a Sputter Coater, and analyzed with SEM at a voltage of 20.0 kV.

Drug loading was assessed by UV–Vis spectrophotometry at 303 nm, previously validated (data not shown). Samples were dissolved in aqueous solution of ethanol 20% (*v*/*v*) and then diluted in water to obtain a MTX concentration of 10 µg·mL^−1^. The mean drug concentration was calculated by the straight-line equation from standard curve fitted plot (*n* = 3).

Fourier Transform Infrared Spectroscopy (FTIR) was performed for pure components, different freeze-dried complexes and respective physical mixtures with the same composition, at room temperature, in the range of 400–4000 cm^−1^, using KBr pellets in a Perkin Elmer^®^ 65 FTIR spectrometer (Perkin Elmer^®^, São Paulo, Brazil).

The carbon, hydrogen and nitrogen for pure components and different freeze-dried complexes were evaluated by elementary analysis in an Elemental Analyzer 2400CHN (Perkin Elmer^®^, São Paulo, Brazil).

Differential scanning calorimetry analysis was carried out in a DSC-50 cell (Shimadzu, Tokyo, Japan), using aluminum pans with lids with about 2 mg of sample, under dynamic nitrogen atmosphere (100 mL·min^−1^), heated at 10 °C·min^−1^ from 25 to 450 °C. The DSC cell was calibrated with indium (melting point 156.5 °C and Δ*H*_fus_ = 28.7 J·g^−1^) and zinc (melting point 419.5 °C) standards.

Thermogravimetry and thermogravimetry derivative curves were obtained with a TGA-50 thermogravimetric analyzer (Shimadzu, Tokyo, Japan), using an aluminum pan with about 5 mg of sample, under dynamic nitrogen atmosphere (50 mL·min^−1^), at a heating rate of 10 °C·min^−1^, from 25 to 450 °C.

X-ray diffraction (XRD) analysis was carried out for pure components, different freeze-dried complexes and respective physical mixtures, using a Rigaku, model Dmax 2500PC X-ray diffractometer (Rigaku, Tokyo, Japan), with a 2θ range between 3° and 55° using Cu*K*α radiation (λ = 1.54 Å). The XRD patterns were recorded under room temperature conditions.

### 3.7. In Vitro Dissolution Studies

*In vitro* dissolution studies were conducted on an USP 30 apparatus 2, with a Hanson SRII 6 Flask Dissolution Test Station (Hanson Research Corporation, Chatsworth, CA, USA). Pure drug, freeze-dried complexes and respective physical mixtures were added in hard gelatin capsules (*n*° = 0) with drug amount equivalent to 10 mg of pure MTX, which were placed in 500 mL of hydrochloric acid solution (HCl 0.1 N, pH = 1.2) at 37 °C, at 50 rpm. At specific intervals, the samples (2 mL) were collected and filtered in a 0.45 μm cellulose acetate membrane, and the mean MTX concentration was determined by UV–Vis spectrophotometry (Thermo Fisher Scientific Inc., Walthan, MA, USA) at 303 nm, which was previously validated (*n* = 3). Different drug dissolution profiles were compared accordingly [56].

### 3.8. Statistics

The analytical results were expressed as mean ± standard deviation (SD). A one-way analysis of variance (ANOVA) was employed as a linearity test and in the comparison of similar experimental results, followed by the *post hoc* test of Dunnet. A *p* value <0.05 was required for significance.

## 4. Conclusions

The present study clearly establishes the interactions of MTX with β-CD in the presence of TEA. Phase solubility studies clearly demonstrate the formation of soluble inclusion complexes with high apparent stability constant (*K*_1:1_). The maximum studied β-CD concentration (15 mM) led to an increase in drug solubility of about fifteen-fold and the association of TEA (0.17–0.67 M) presented a synergistic effect, which enhanced by thirty-fold. This effect was clearly explained by the NMR study, in which the 2D ROESY experiments showed the interaction between the aromatic protons of MTX and H_3_, H_5_ and H_6_ protons of β-CD, indicating that the aromatic ring III of the drug is deeply internalized into the cyclodextrin cavity in both binary and ternary complexes. Molecular modeling studies supported this inclusion complex formation and contributed to the explanation of how these components interact in a ternary MTX:β-CD:TEA complex, in which TEA significantly increases MTX affinity for β-CD compared with the binary complex. In contrast to other studies, TEA did not compete with the CD cavity, but it stabilized the MTX-β-CD interaction. The experimental results from host–guest interaction were used to obtain ternary MTX:β-CD:TEA freeze-dried complexes. FTIR studies demonstrated that the drug–CD interactions identified by NMR studies remained in solid phase. Thermal and structural studies also indicated this perspective, mainly regarding the characteristic increment of drug thermal stability. The success of ternary complex for the purposed aim was also demonstrated in the *in vitro* dissolution studies.
